# Commentary: Tolvaptan for Autosomal Dominant Polycystic Kidney Disease (ADPKD) - an update

**DOI:** 10.1186/s12882-025-03960-4

**Published:** 2025-02-14

**Authors:** Matt Gittus, Helen Haley, Tess Harris, Sarah Borrows, Neal Padmanabhan, Danny Gale, Roslyn Simms, Terri Williams, Aaron Acquaye, Alisa Wong, Melanie Chan, Eduardo Lee, Albert CM Ong

**Affiliations:** 1 University of Sheffield, Sheffield, United Kingdom; 2 Sheffield Teaching Hospitals NHS Trust, Sheffield, United Kingdom; 3 University Hospitals of North Midlands, Birmingham, United Kingdom; 4 PKD Charity, London, United Kingdom; 5 Queen Elizabeth Hospital Birmingham, Birmingham, United Kingdom; 6 NHS Greater Glasgow and Clyde, Glasgow, United Kingdom; 7 Royal Free London NHS Foundation Trust, London, United Kingdom; 8 Hull and East Yorkshire Hospitals NHS Trust, Hull, United Kingdom; 9 Imperial College Healthcare NHS Trust, London, United Kingdom; 10 Guy’s and St Thomas’ NHS Foundation Trust, London, United Kingdom

**Keywords:** Autosomal Dominant polycystic kidney disease, ADPKD, Tolvaptan

## Abstract

Autosomal Dominant Polycystic Kidney Disease (ADPKD) affects up to 70 000 people in the UK and the most common inherited cause of end-stage kidney disease (ESKD). It is generally a late-onset multisystem disorder characterised by bilateral kidney cysts, liver cysts and an increased risk of intracranial aneurysms. Approximately 50% of people with ADPKD reach ESKD by age 60. Disease-associated pain, discomfort, fatigue, emotional distress and, impaired mobility can impact health-related quality of life. The approval of tolvaptan, a vasopressin V2 receptor antagonist, has greatly advanced the care for people with ADPKD, shifting the focus from general chronic kidney disease management to targeted therapeutic approaches. While guidance from NICE and SMC provides a foundational framework, this is not clear or comprehensive enough to offer practical guidance for healthcare professionals in real-world settings. This commentary expands on the previous United Kingdom Kidney Association (UKKA) commentary in 2016 with an updated evidence base, the incorporation of real-world data and expert opinion to provide practical guidance to healthcare professionals. Through co-development with people affected by ADPKD, it now incorporates valuable patient perspectives and offers practical recommendations for the UK kidney community seeking to harmonise the quality of care of all people with ADPKD.

## Background

Autosomal Dominant Polycystic Kidney Disease (ADPKD) is the most common hereditary kidney disease with an estimated prevalence ranging from 1 in 1000 to 1 in 2500 [1–5]. Variants in two genes, *PKD1* and *PKD2*, encoding for polycystin-1 or polycystin-2 account for 75–85% and 15–25% of genetically resolved cases respectively [6–10]. Reduced polycystin function leads to increased cyclic adenosine monophosphate signalling, which is a key driving mechanism for cyst growth and fluid secretion [11, 12]. ADPKD is generally a late-onset multisystem disorder characterised by bilateral kidney cysts, liver cysts and an increased risk of intracranial aneurysms. Less common manifestations include pancreatic cysts, seminal vesicle cysts, and mitral valve prolapse amongst others [13]. Progressive cyst development and growth ultimately leads to kidney dysfunction with approximately 50% of people requiring kidney replacement therapy by age 60 [14]. ADPKD can impose a significant burden on health-related quality of life due to pain, discomfort, fatigue, emotional distress and, limiting daily activities [15–18].

Tolvaptan is a short-acting vasopressin 2 receptor antagonist that blocks vasopressin signalling leading to a reduction in cAMP in cystic kidney tissues [19]. The landmark Tolvaptan Efficacy and Safety in Management of Autosomal Dominant Polycystic Kidney Disease (TEMPO) 3:4 trial, conducted between 2007 and 2012, represented a major breakthrough in the management of Autosomal Dominant Polycystic Kidney Disease (ADPKD) [20]. This trial supported the approval of tolvaptan by the National Institute for Health and Care Excellence (NICE) and the Scottish Medicines Consortium (SMC) for people with rapidly progressing ADPKD [21, 22]. NICE recommended starting treatment at CKD stages 2–3 [21], while SMC allowed initiation from stages 1–3 [22]. As of February 2023, Otsuka reports that tolvaptan has been licensed in over 43 countries including Australia, the European Union, Japan, and the United States, among others.

Since the 2016 commentary by the UKKA, there have been significant developments including clinical trials [23, 24], post-hoc analysis [25], risk assessment tools [26, 27], broader access to genetic testing, and availability of real-world data, all of which warrant an update to incorporate these advances. For this reason, the following recommendations were prepared on behalf of the UKKA working group for the management of ADPKD with tolvaptan and endorsed by the UKKA.

## An update on the efficacy of tolvaptan in ADPKD

In 2017, the TEMPO 4:4 study, an open-label extension of TEMPO 3:4, examined the long-term effects of tolvaptan over 24 months. While tolvaptan effectively maintained kidney function, the reduction in total kidney volume (TKV) was not sustained, possibly due to the non-randomised design and unadjusted baseline characteristics in the study [23]. The Replicating Evidence of Preserved Renal Function: An Investigation of Tolvaptan Safety and Efficacy in ADPKD (REPRISE) trial enrolled patients aged 18–55 with baseline estimated glomerular filtration rate (eGFR) 25–65 ml/min/1.73m^2^ and those aged 56 to 65 years old with a baseline eGFR 25–44 ml/min/1.73m^2^. At 1-year follow-up, tolvaptan slowed eGFR decline by 1.27 ml/min/1.73m^2^ in all groups except those aged over 55 years and, with early-stage CKD [28, 29]. Evidence for the effectiveness of tolvaptan among people from ethnic backgrounds other than white was limited; only 4% of study participants in TEMPO 3:4 were described as black compared to over 80% described as white [20]. Further research is needed in people from black and Hispanic ethnic backgrounds since they are reported to reach kidney failure earlier and are less likely to receive a pre-emptive kidney transplant compared to people from a white ethnic background [30].

A retrospective analysis from an expert centre (Mayo Clinic) provided up to 11 years long-term follow-up for 97 people with ADPKD treated with tolvaptan (median 4 years, range 1.1–11.2 years). When compared to matched controls from other studies, the predicted eGFR decline mirrored results from TEMPO 3:4 and REPRISE [29]. Finally, secondary analysis of TEMPO 3:4 showed additional benefits to slowing the decline in kidney function, with a reduction in kidney pain events and urinary tract infections compared to placebo [31].

## Practical guide for prescribing tolvaptan

The following steps outline the essential considerations and processes for safely prescribing tolvaptan in the management of ADPKD. These steps have been adjusted from a practical guide published by Chebib et al. [31].

A. Ensure a confirmed diagnosis of ADPKD.
B. Confirm eligibility for tolvaptan.
C. Confirm evidence of rapidly progressive disease or high risk of progression.
D. Confirm that there are no exclusions.
E. Consider potential drug interactions.
F. Initiate and titrate Tolvaptan.
G. Establish monitoring plan.
H. Manage side effects and adverse effects associated with tolvaptan.

### Ensure a confirmed diagnosis of ADPKD

*Recommendation 1*: *We recommend that all people with ADPKD being considered for tolvaptan should have an established diagnosis of ADPKD through diagnostic imaging and/or genetic testing.*

#### Diagnostic imaging

In atypical cases or when ultrasound findings don’t align with clinical symptoms, magnetic resonance imaging (MRI) and computed tomography (CT) offer greater sensitivity for detecting small cysts (< 5 mm) [32, 33]. In the absence of family history, detecting ≥ 10 cysts per kidney with any imaging modality can be considered diagnostic for ADPKD, in the presence of bilaterally enlarged kidneys and the exclusion of other forms of cystic kidney diseases [34].

#### Genetic testing

Whole genome sequencing is available through the National Health Service (NHS) Genomic Medicine Service in England [35], the Scottish Strategic Network for Genomic Medicine in Scotland [36], the All Wales Medical Genomics Service in Wales [37] and Belfast City Hospital Clinical Genetics Department in Northern Ireland [38]. Identifying a monoallelic pathogenic variant in a cystic kidney disease gene (most commonly *PKD1* or *PKD2*) can confirm a diagnosis of ADPKD. This is particularly useful in the context of atypical disease or those without a family history (~ 30%). A genetic diagnosis has several clinical applications including risk assessment, family planning, and confirming kidney donor suitability [17].

### Confirm eligibility for tolvaptan

#### Age at initiation

*Recommendation 2*: *We recommend that people with ADPKD being considered for tolvaptan should be aged 18 and above at the time of treatment initiation.*

The TEMPO 3:4 and REPRISE trials included participants aged 18-50 [20] and 18-65 [24], respectively. Currently, no large-scale randomised controlled trials have evaluated the efficacy and safety of tolvaptan in children and adolescents. A phase 3b study of participants aged 4-17 showed a non-significant reduction in height-adjusted annual increase total kidney volume (TKV) and eGFR slope after 12 months of tolvaptan compared to placebo [39]. While there is insufficient data to support tolvaptan use in those under 18, no upper age limit is recommended but age-related kidney function should be considered (see Table 1) [40].

**Table 1:** Average measured eGFR by age group in people without CKD from the National Kidney Foundation [41]

Age group (years)	Average eGFR (ml/min/1.73m^2^)
20–29	116
30–39	107
40–49	99
50–59	93
60–69	85
70 +	75

#### CKD Stage at initiation

*Recommendation 3*: *In England and Wales we recommend initiating tolvaptan in individuals at CKD stage 2–3 (30–89 ml/min/1.73m*^*2*^*). In Scotland*,* we recommend initiating tolvaptan in individuals at CKD stages 1–3 (≥ 30 ml/min/1.73m*^*2*^*).*

Kidney function measurements should be confirmed by two blood tests, at least 72 h apart, and without intercurrent illness. In contrast to the SMC, NICE excludes patients with stage 1 CKD (eGFR ≥ 90 ml/min/1.73m^2^) due to an unfavourable cost-benefit analysis and non-significant slope in this subgroup although there was a significant reduction in TKV increase [21]. Serial evaluation for patients with stage 1 CKD is recommended, reviewing symptoms and family history, as this subgroup may still experience rapid disease progression.

### Confirm evidence of rapidly progressive disease or high risk of progression

**Fig. 1:**
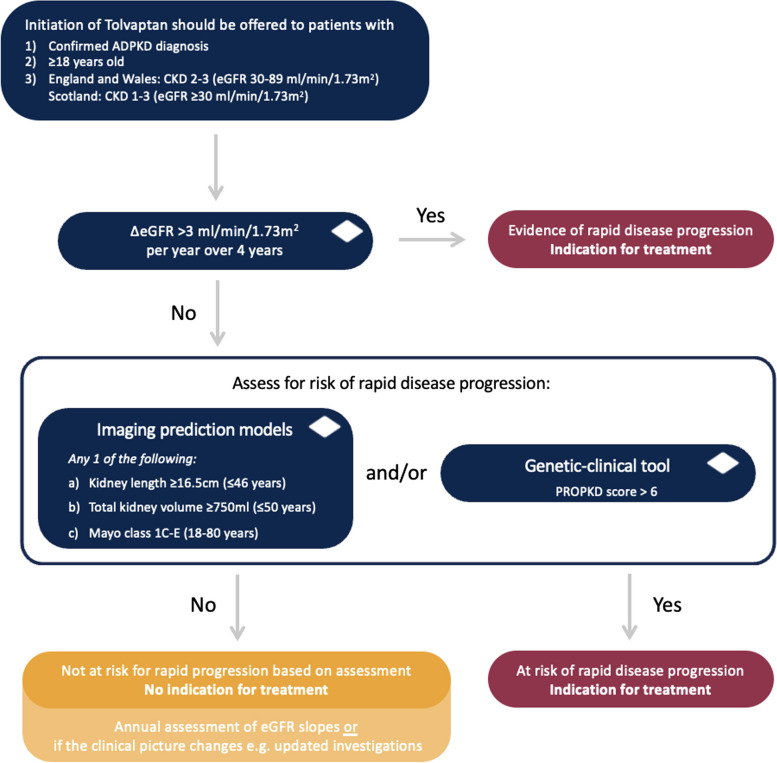
Approach to risk assessment for rapid disease progression

Both NICE and SMC require evidence of “rapidly progressing disease” for tolvaptan initiation, but neither defines this term explicitly. The primary method of assessing rapid progression is through annual changes in eGFR. However, because compensatory glomerular hyperfiltration can mask a decline in eGFR in early stages of ADPKD [13, 42], eGFR alone may not capture early kidney damage [13, 43]. Identifying those at risk of rapid progression, even with normal or near-normal kidney function, is critical for timely intervention, including the use of tolvaptan, and enhanced monitoring. An approach to risk assessment is outlined in Fig. 1.

## Defining rapidly progressive disease

*Recommendation 4*: *We recommend that a definition for evidence of rapid disease progression and eligibility for tolvaptan is a sustained decline in eGFR of ≥ 3 ml/min/1.73m*^*2*^*per year (at least 5 measurements over 4 years).*

Progression in ADPKD can vary significantly within and between families [11]. Historical eGFR trends offer the best indication of disease progression. Irazabal et al. (2015) demonstrated that typical annual eGFR declines are 2.63 ml/min/1.73m^2^ for men and 2.43 ml/min/1.73m^2^ for women through their modelling of an imaging-based risk classification [26]. The TEMPO 3:4 and REPRISE trials reported an average annual eGFR decline of 3.5 ml/min/1.73m^2^ [20, 24]. For this reason, we recommend defining rapid progression as an annual decline in eGFR of ≥ 3 ml/min/1.73m^2^ per year over 4 years, based on at least 5 measurements. Multiple measurements are required to account for the natural day-to-day fluctuations in eGFR [44] and the small subset of people with ADPKD that exhibit non-linear eGFR losses [45]. The previous commentary recommended ≥ 5 ml/min/1.73m^2^ annual decline but this acute decline is rarely seen amongst people with ADPKD in clinical practice and other acute causes should be considered [20, 23, 24].

There are specific considerations when assessing kidney function in certain populations. In people who may become pregnant, baseline kidney function should be evaluated at least three months post-partum as pregnancy itself can lead to physiological changes in eGFR [46]. In elderly people with suspected rapid eGFR decline, it is important to exclude causes unrelated to ADPKD [40].

## Markers of high risk of disease progression

*Recommendation 5*: *We recommend assessment for risk of disease progression if there is a lack of evidence for rapid disease progression based on eGFR criteria. This evidence can be based on diagnostic imaging and/or genetic testing. It can be determined by (a) kidney length ≥ 16.5 cm (≤ 46 years)*,* (b) total kidney volume ≥ 750 ml (TKV) (≤ 50 years)*,* (c) Mayo imaging Class 1-E*,* (d) PROPKD score > 6.*

People with ADPKD who do not have evidence of rapidly progressing disease based on eGFR losses should be evaluated for their risk of rapid disease progression using imaging, clinical and genetic prognostic markers. These markers, which are associated with more severe disease trajectories, can help predict future kidney outcomes before any noticeable decline in eGFR [47, 48].

### Risk prediction using imaging modalities

*Kidney length*: The Consortium for Radiologic Imaging Study of PKD (CRISP) studies found that a kidney length of 16.5 cm (measured by US or MRI) effectively predicts the progression to CKD stage 3a over 8 years in participants younger or equal to 46 years. Ultrasound is preferred due to wider availability, lower cost and fewer restrictions in terms of metallic objects/implanted devices [49]. However, kidney length cannot reliably differentiate between *PKD1* or *PKD2* variants and its use alone may misclassify people with atypical ADPKD which is often focal but with a smaller number of large kidney cysts [50]. A longitudinal study reported that US height-adjusted mean kidney length > 9.5 cm/m combined with a *PKD1* truncating variant, yielded a 100% positive predictive value for rapid progression (annual eGFR decline > 2.5 ml/min/1.73m^2^) and kidney failure before age 60 [51]. Thus, we recommend that an average kidney length greater than 16.5 cm in those aged 46 or younger can be regarded as evidence of a high risk of progression, in the knowledge that the best use of kidney length as a predictor of rapid progression is in those with typical ADPKD.

*Total kidney volume*: Extended CRISP study findings demonstrated that a TKV ≥ 600 ml/m predicts the risk of developing ESKD [52]. Trials like TEMPO 3:4 and REPRISE used a TKV ≥ 750 ml as an inclusion criterion for participants aged ≤ 50 years [20, 24]. Furthermore, Japan has adopted a TKV ≥ 750 ml as an approved indication for tolvaptan treatment [53]. Therefore, we recommend that a TKV ≥ 750 ml can be used as a marker of disease burden and high risk of disease progression in individuals aged 50 years or younger. The previous commentary included > 5% change in TKV with 3 measurements over 2–3 years but since this is rarely used in clinical practice in the UK, it has not been included in this commentary.

*Mayo imaging classification*: When MR or CT imaging is performed, the Mayo Imaging Classification (MIC) category can be determined using TKV, adjusted for age and height. MIC has been validated as a sensitive prognostic marker for people with “typical” ADPKD aged 15–80, helping predict progression to ESKD [26]. People classified as class 1 C-E are considered to be at “high risk” of rapid progression. If height measurements are not available, a single TKV measured by MR imaging ≥ 750 ml can be used as a predictor of rapid disease progression in people with ADPKD aged 18–50 years [11]. People with ADPKD classified as “typical” ADPKD class 1 A-B, “atypical” ADPKD class 2 and TKV < 750 ml are considered to have a low risk of progression [26].

## Risk prediction using genetic and clinical factors

*Predicting Renal Outcomes in ADPKD (PROPKD) score*: The pathogenic variant in ADPKD can predict disease progression, with protein truncating variants in *PKD1* linked to early ESKD with a median age 55 compared to missense *PKD1* and *PKD2* variants with median ages 67 and 79 respectively. The PROPKD score integrates genetic and clinical factors to identify those at risk of early-onset ESKD. A score of < 4 indicates low risk, while scores > 6 suggest high risk for rapid disease progression [27].

### Confirm that there are no exclusions

*Recommendation 6*: *We recommend that people with ADPKD should be assessed for potential contraindications/precautions prior to tolvaptan initiation.*

*Recommendation 7*: *We do not recommend tolvaptan use in people who are pregnant or breast-feeding.*

*Recommendation 8*: *We recommend advising people who may become pregnant of the potential teratogenic risk of tolvaptan and encourage them to use contraception.*

Prior to prescribing tolvaptan, it is essential to discuss specific information with patients, as outlined in recommendations by Otsuka. Below is a summary of the contraindications and precautions associated with comorbidities that should be considered [54].

## Contraindications

Tolvaptan should not be prescribed if the patient presents with any of the following conditions or signs [54]:

- Elevated liver enzymes
- Hypersensitivity to the active substance or any of its constituents
- Volume depletion
- Uncorrected hypernatraemia (> 145 mmol/L)
- Inability to perceive or respond to thirst
- Pregnancy
- Breastfeeding
- Anuria

## Precautions

If any of the following apply to the patient then tolvaptan may be prescribed with caution along with appropriate monitoring [54]:

- Severe hepatic impairment (Child-Pugh class C)
- Cirrhosis
- Limited access to water
- Dehydration
- Partial obstruction of urinary outflow
- Fluid and electrolyte imbalance
- Serum sodium abnormalities
- Anaphylaxis
- Lactose and galactose intolerance
- Diabetes mellitus
- Elevated uric acid concentration
- Use of medicines likely to interact with Tolvaptan

## Specific considerations in relation to pregnancy

Tolvaptan has been demonstrated to be teratogenic in animal models at maternally toxic doses, approximately 1-4 times the recommended dose of 90 mg/30 mg once daily. Thus, Otsuka recommends that tolvaptan is contraindicated during pregnancy [54, 55]. They recommend using contraception for at least four weeks before starting and for four weeks after stopping tolvaptan. We recommend that people who may become pregnant should be advised of the risks and encouraged to use contraception [55].

## Specific considerations in relation to breastfeeding

It is not known whether tolvaptan is excreted in human milk, what effects there are on the breast-fed infant or the effects on milk production. Animal studies have shown the excretion of tolvaptan in milk [55]. Otsuka advises people not to breastfeed while taking tolvaptan and to delay breastfeeding for four weeks after stopping tolvaptan [56].

### Consider potential drug interactions

*Recommendation 9*: *We recommend that special care should be given when prescribing Tolvaptan alongside drugs that interfere with the action of CYP3A4.*

*Recommendation 10*: *We recommend tolvaptan dose adjustment should be considered with concurrent use of strong or moderate CYP3A4 inhibitors but not with CYP3A4 inducers.*

The British National Formulary (BNF) lists the drug interactions that can occur with concurrent use of tolvaptan. Tolvaptan is metabolised by the microsomal P450 drug-metabolising enzyme known as CYP3A4, so tolvaptan levels can be influenced by inhibitors and inducers of this enzyme [57].

Medications can interact with tolvaptan with four main consequences:

- Increase exposure to tolvaptan
- Decrease exposure to tolvaptan
- Increased risk of hyperkalaemia
- Tolvaptan increases exposure to other medication

#### Increase exposure to tolvaptan (CYP3A4 inhibitors)

Otsuka advises reducing the dose of tolvaptan with concurrent use of strong and moderate CYP3A4 inhibitors [55]. The advised adjustments are indicated in Table 2 [58].

**Table 2:** Tolvaptan dose adjustments for strong and moderate CYP3A4 inhibitors [58, 59]

		Dose adjustment	
		Strong inhibitors	Moderate inhibitors
**Total daily dose**	**120 mg** **(90 mg + 30 mg)**	30 mg once daily (potential reduction to 15 mg)	60 mg twice daily (45 mg + 15 mg)
	**90 mg** **(60 mg + 30 mg)**	30 mg once daily (potential reduction to 15 mg)	45 mg twice daily (30 mg + 15 mg)
	**60 mg** **(45 mg + 15 mg)**	15 mg once daily	30 mg twice daily (15 mg + 15 mg)

#### Decrease exposure to tolvaptan (CYP3A4 inducers)

Otsuka does not advise dose adjustment of tolvaptan with concurrent use of strong or moderate CYP3A4 inducers [59].

#### Increased risk of hyperkalaemia

Tolvaptan is associated with an acute reduction of extracellular fluid volume which could result in increased serum potassium levels [55, 60]. Certain drugs such as angiotensin converting enzyme inhibitors (ACEi), angiotensin receptor blockers (ARB) or aldosterone antagonists can further increase the risk of hyperkalaemia when combined with tolvaptan. There is no published evidence on the optimal timing for monitoring of hyperkalaemia when co-administering tolvaptan with other medications that increase the risk of hyperkalaemia. It is advisable to conduct earlier monitoring, such as one week after initiation, when prescribing multiple medications that may affect potassium levels [61].

#### Tolvaptan influences the effect of other medications

Tolvaptan may increase the effects of the following medications: digoxin, dabigatran, sulfasalazine and metformin. It may lower the effect of desmopressin, a vasopressin analogue, used to increase clotting factors or control urine output/bedwetting [62].

### Initiate and titrate dose of tolvaptan

*Recommendation 11*: *We suggest a starting dose of tolvaptan 45 mg in the morning and 15 mg 6-8 h after the first dose.*

*Recommendation 12*: *We suggest doses should be up-titrated based on healthcare professional preference. Some centres increase doses at 28 day intervals in accordance with standard pack sizes of tolvaptan.*

*Recommendation 13*: *We suggest titrating to a maximum dosage of 120 mg per day Tolvaptan (90 mg/30 mg) in all patients unless not tolerated or contraindicated.*

Tolvaptan in the management ADPKD is administered as a split dose due to its half-life, with a maximum daily dose of 120 mg. This dosing is based on the TEMPO 3:4 trial where 90 mg/30 mg was the highest tolerated dose [20]. Tolvaptan is available in 15 mg, 30 mg, 60 mg and 90 mg preparations [55].

The recommended initial dosing regimen for tolvaptan is 60 mg per day, divided into 45 mg in the morning and 15 mg 6–8 h later. To minimise nocturnal symptoms, patients are advised to take the final dose by 5pm [55]. For individuals with non-traditional working hours, these timings should align with their waking schedule while maintaining the specified interval between doses.

**Fig. 2:**
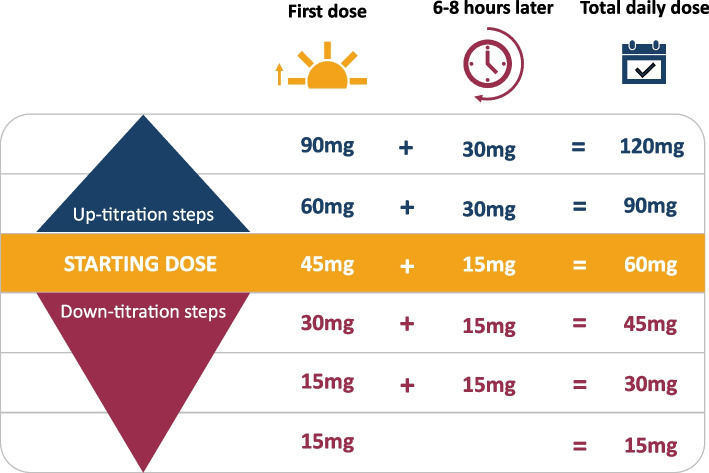
Dosing regime and titration steps for tolvaptan

We agree with the manufacturer’s recommendation that the aim should be to up-titrate tolvaptan dose to a maximum of 120 mg (90 mg and 30 mg). In the TEMPO 3:4 trial, tolvaptan was started at 45 mg/15 mg which was up-titrated weekly to 60 mg/30 mg and then 90 mg/30mg [20]. There is no published guidance on the optimal titration regimen with varied practices between kidney units. We recognise the benefits of a pragmatic approach, such as up-titrating the dose of tolvaptan every four weeks based on the 28 tablet pack size of tolvaptan, with adjustments based on patient tolerance. In cases where people taking tolvaptan are not able to tolerate the starting dose of 60 mg daily (45 mg/15 mg), a dose reduction can be considered as an alternative to discontinuation. An example of the approach to titration is summarised in Fig. 2.

According to the Summary of Product Characteristics (SPC) for tolvaptan, co-administration with a high-fat meal may increase the peak concentration of tolvaptan, although this effect has only been established for the 90 mg dose and not for 30 mg or 60 mg doses [63]. While this food effect is generally considered not clinically significant, the manufacturer recommends that the morning dose be taken under fasting conditions, specifically 30 min prior to breakfast. However, other clinical resources do not have this recommendation (BNF [59], Martindale through Medicines Complete [64] and UpToDate Lexidrug [65]). Furthermore, this morning timing in relation to food was not a component of clinical trials. Given the potential impact on patients who may already experience disturbed sleep due to nocturnal aquaretic effects, a pragmatic approach is recommended. We suggest that patients may take the morning dose with or without food, provided they are not consuming a very high-fat meal e.g. a full English breakfast.

### Establish a monitoring plan during tolvaptan

#### Monitoring response to tolvaptan

*Recommendation 14*: *We do not recommend any specific monitoring for tolvaptan treatment response or efficacy.*

There are no validated markers to monitor or predict the effect of tolvaptan on eGFR or TKV at an individual level. V2 receptor blockade can be assessed by measuring urine osmolality, but while this reflects adherence, it is not a reliable efficacy marker due to fluid intake variability [66]. Copeptin increases with tolvaptan use, showing potential as a biomarker, but current data is insufficient for clinical application [67]. Comparisons of changes in eGFR and TKV to pre-treatment trends or MIC categories lack validated sensitivity so are not recommended for individual monitoring [20, 23, 24].

#### Monitoring kidney function and electrolytes

*Recommendation 15*: *We recommend measuring kidney function monthly in line with liver function monitoring. An initial decline of 3–9% in eGFR may be expected when tolvaptan is started which is reversible on cessation.*

*Recommendation 16*: *We recommend the timing of the decision to stop tolvaptan when approaching kidney failure is best made between the person with ADPKD and their responsible healthcare professional.*

*Recommendation 17*: *Following the initiation of kidney replacement therapy*,* we recommend that tolvaptan should be stopped.*

An initial decline of 3-9% in eGFR may occur after starting tolvaptan, depending on baseline kidney function which is reversible upon cessation [20, 23, 24]. This is similar but less pronounced than with angiotensin-converting enzyme inhibitors (ACEi) or angiotensin receptor blockers (ARBs) [68, 69]. Kidney function is often monitored at least on the same schedule as liver function tests – monthly for the first 18 months then every 3 months thereafter.

Hyperkalaemia can occur with tolvaptan due to the acute reduction in extracellular fluid volume. In general, we suggest following the usual monitoring regime for kidney function monthly. Earlier monitoring at one week after initiation may be advisable where there is a higher risk of hyperkalaemia. Examples include the prescribing of multiple medications that influence potassium levels, CKD stage 3 or higher, aged 60 years or older, certain comorbidities (such as diabetes mellitus or peripheral arterial disease), or a history of hyperkalaemia [61].

The REPRISE trial demonstrated tolvaptan efficacy down to an eGFR of 25 ml/min/1.73m^2^ [24]. While there are no trials below this threshold, the BNF advises stopping tolvaptan at CKD stage 5 (eGFR < 15 ml/min/1.73m^2^) [59]. We recommend discontinuing tolvaptan when kidney replacement therapy is initiated. The decision to stop tolvaptan earlier as eGFR declines is best made through shared decision-making taking patient preferences, healthcare professional experience and fluid balance considerations into account.

#### Monitoring liver function

*Recommendation 18*: *We recommend measuring liver function monthly during the first 18 months of treatment then 3 monthly afterwards.*

Liver function tests are mandated prior to the initiation of tolvaptan and monthly for 18 months then 3 monthly after that. This is based on the finding of the REPRISE trial that nearly all cases of treatment-associated liver abnormalities occurred within the first 18 months [24]. Advice on managing elevated liver enzymes is covered in the drug-induced liver injury section.

### Manage side effects and adverse effects

*Recommendation 19*: *We recommend discussing side effects with patients and providing written patient information prior to initiating tolvaptan.*

*Recommendation 20*: *We recommend withholding tolvaptan during periods of acute illness due to the increased risk of dehydration. This can be described as “sick day guidance” as advised for ACEi/ARBs*.

*Recommendation 21*: *In the event of suspected side effects or adverse drug reactions*,* we recommend that all healthcare professionals should submit a report to the Yellow Card Scheme.*

Tolvaptan is associated with common and rare side effects, with clinical trials and real-world data indicating an increase in side effect frequency and severity at higher doses [20, 24, 70, 71]. All healthcare professionals involved in patient care have a responsibility to report suspected adverse drug reactions or new side effects. This is to provide an early warning that the safety of a product may require further investigation. Reports can be made using the yellow card website or on the Yellow Card app [72].

The main side effects of tolvaptan and their reported frequencies are summarised in Table 3 based on the study by Raina et al. [70] and the Teva Pharmaceuticals patient information leaflet for tolvaptan [56].

**Table 3:**
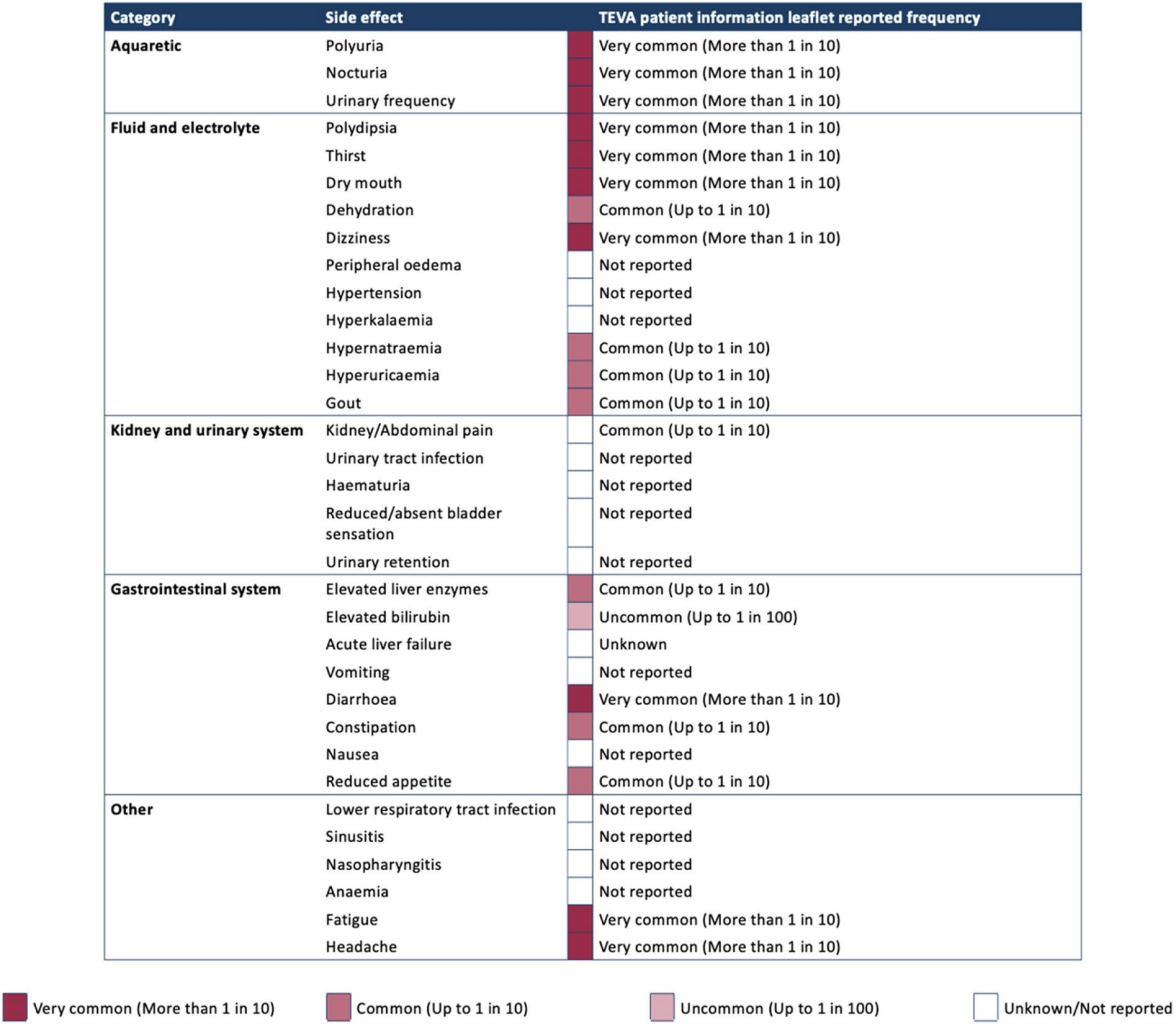
Side effects for tolvaptan by category and reported frequency [56, 70]

## Aquaretic side effects

As a selective V2 vasopressin receptor antagonist, it competes with vasopressin to bind to the V2 receptor preventing the translocation of aquaporin-2 channels in the kidney collecting duct leading to water excretion [73]. As a result, people taking tolvaptan commonly experience aquaretic side effects such as polyuria, nocturia, pollakiuria, polydipsia, thirst, dehydration and dry mouth [13, 74].

While people with early stage ADPKD stand to benefit most from tolvaptan, they are also more likely to experience these aquaretic side effects due to their relatively better urine concentrating ability compared to those with later stage disease. Thus, they will likely experience more aquaretic side effects reducing tolerability [75].

Some measures may make the aquaretic side effects more manageable:

- Time-dependent attenuation
- Dietary changes
- Pharmacological

### Time-dependent attenuation

People with ADPKD are advised to start tolvaptan on a weekend or when not at work, to allow time for adjustment to the aquaretic effects [31]. To minimise nocturia and sleep disturbance, we recommend that the second dose of tolvaptan not be taken too late in the afternoon. If nocturia continues to be a significant issue despite these adjustments, a reduction in the second dose may be considered, though this could reduce the overall efficacy of the treatment.

### Dietary changes

Dietary changes that decrease osmolar loads, such as reducing salt and protein intake, may alleviate some of the aquaretic effects of tolvaptan [76, 77]. A lower sodium intake can help reduce natriuresis with the added benefit of improved blood pressure control [75, 77]. High-sodium foods, such as tinned items, instant mixes, condiments, snacks, pre-prepared meals, soft drinks and fast food, should be avoided. Furthermore, salt intake has been associated with accelerated disease progression in ADPKD [78]. We suggest people with ADPKD consume less than 5 g of sodium daily [77–79].

In CKD management, excessive protein consumption is discouraged, with KDIGO guidelines recommending 0.8 g/kg/day for adults with an eGFR < 30 ml/min/1 [80]. Theoretically, the lower solute load from a low protein diet may reduce aquaretic side effects but this has not been shown in clinical trials. Studies on protein restriction in people with ADPKD in general, have failed to show any benefits and demonstrated a trend towards increased morbidity at low eGFR levels [78].

The effects of caffeine on natriuresis and diuresis in the context of ADPKD and tolvaptan is not fully understood. Coffee with high caffeine content can induce an acute diuretic effect [81]. Theoretically this may add to the aquaretic effects of tolvaptan but this has not been studied. The effect of caffeine on disease progression has been studied but remains inconclusive. Animal studies have shown that caffeine intake increases cyst growth through phosphodiesterase inhibition and cAMP accumulation [82]. However, caffeine has not been demonstrated to have a significant detrimental effect on disease progression in humans [83–85]. At this time, we suggest avoiding excessive caffeine intake, especially later in the day due to the potential impact on sleep quantity and quality.

### Pharmacological

Thiazide diuretics have been demonstrated to be effective in reducing polyuria by up to 50% in people with nephrogenic diabetes insipidus; the mechanism behind this paradoxical antidiuretic effect has not been fully elucidated [86]. A small-scale trial has suggested that the mechanism of action of thiazide diuretics may be an option to improve the tolerability of tolvaptan and adherence in the management of ADPKD [87]. At present there is insufficient evidence to recommend their use.

### Dehydration

*Recommendation 22*: *We suggest that a clinical assessment should be performed prior to initiation to identify risk factors for dehydration*,* fluid retention and/or dilution hyponatraemia.*

*Recommendation 23*: *We suggest that people with ADPKD*,* normal thirst and an eGFR > 30 ml/min/1.73m*^*2*^*should be informed of the increased need for hydration throughout the day and to ensure regular access to fluids.*

*Recommendation 24*: *We suggest that people with ADPKD and an eGFR < 30 ml/min/1.73m*^*2*^*or those with a clinical contraindication to high fluid intake should drink to thirst and/or follow individualised clinical advice.*

Given the need for high compensatory water intake before starting tolvaptan, people with ADPKD should be assessed for potential fluid intake issues and ability to meet the increased fluid requirements alongside their lifestyle and existing comorbidities. People with a compromised capacity to perceive and communicate thirst may be at higher risk of dehydration and hypernatremia. Otsuka advises healthcare professionals to instruct people taking tolvaptan to water when thirsty, and throughout the day and night if awake [55]. In clinical practice, the advice often given to people with ADPKD taking tolvaptan is to match their intake to their urine output, but this can be difficult to measure in practice. Target daily fluid intake volumes are often given but there is no clear evidence for any specific target volume. Best practice would be to personalise fluid intake for individual patients. In cases of reduced kidney function (< 30 ml/min/1.73m^2^) or comorbidities such as heart failure, we suggest that people on tolvaptan should be advised to drink to thirst or have a lower fluid intake recommendation due to the risk of fluid overload.

People with ADPKD and their healthcare providers should be advised to withhold tolvaptan and increase hydration in the setting of acute illnesses or high insensible fluid losses (e.g. warm weather) to prevent dehydration [31, 88]. This follows “sick day guidance” similar to that recommended for medications like ACEi and ARBs [89]. Tolvaptan should resume, at the dose prior to withholding, 24 h after recovery from the acute illness. Tolvaptan should also be withheld 24–48 h before elective surgery and not resume until patients are able to maintain adequate hydration [31].

Prescribers should be cautious when combining tolvaptan with other medications that increase the risk of hypovolaemia, such as diuretics. It may be appropriate to consider the need and suitability for both medications due to the increased risk of hypovolaemia, hypernatremia and kidney injury. Additionally, although the interaction between tolvaptan and sodium-glucose transport protein 2 (SGLT2) inhibitors is not well studied, there is a theoretical risk of increased diuretic effects and glomerular haemodynamic changes leading to a reduction in eGFR when both medications are used together [90].

## Drug-induced liver injury

*Recommendation 25*: *We recommend informing people taking tolvaptan of the risk of liver injury and encouraging them to self-report symptoms.*

*Recommendation 26*: *We recommend withholding tolvaptan in the event of suspected drug-induced liver injury to allow time to exclude other causes. It is important to continue monitoring liver enzymes until they return to normal or the individual’s baseline.*

Idiosyncratic drug-induced liver injury (DILI) can occur after tolvaptan exposure, ranging from asymptomatic elevation in liver enzymes to acute liver failure [91]. People taking tolvaptan should be informed of the potential signs and symptoms of liver injury, as these may develop between clinic visits. We recommend that best practice would be for healthcare teams to review patients with signs or symptoms of liver injury within 48 h.

**Fig. 3:**
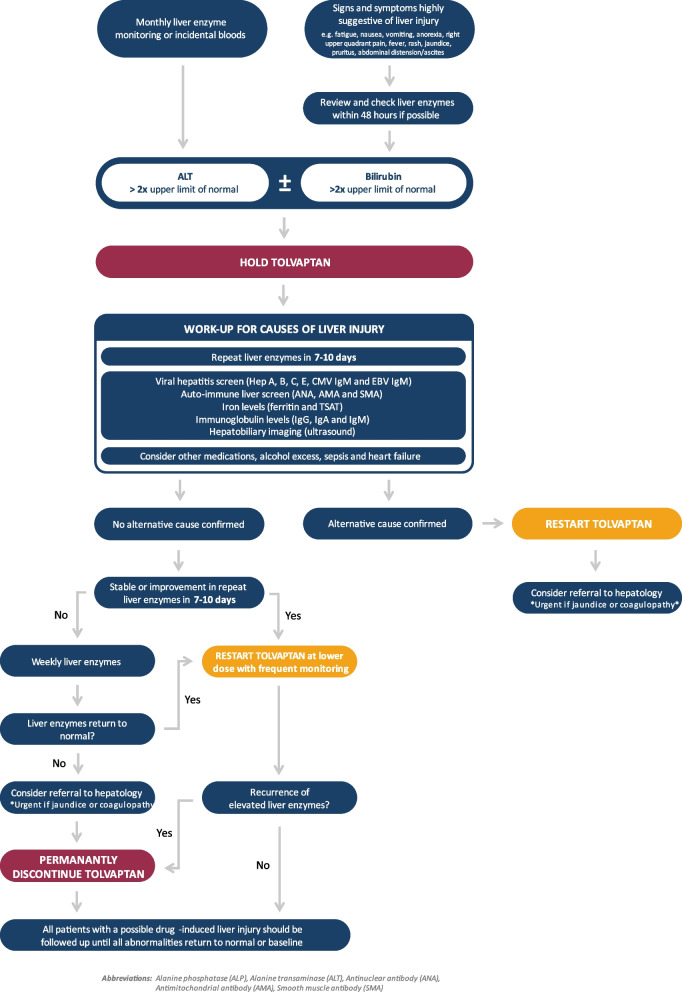
Recommendations for evaluation and management of suspected drug-induced liver injury (adapted from Chebib et al. [31] and British Society of Gastroenterology [94])

In the TEMPO 3:4 trial, DILI was identified as a rare side effect with 1.2% of participants discontinuing the trial for this reason [20]. In the extension TEMPO 4:4 trial, liver enzyme levels were monitored monthly for the first 18 months, during which no severe DILI cases were reported [23]. It is important to note that elevated liver enzymes do not always correlate with the extent of liver damage [92]. DILI is commonly defined using adjusted Hy’s law thresholds, characterised by alanine transferase (ALT) levels exceeding three times upper limit of normal and/or bilirubin levels exceeding twice the upper limit of normal in the absence of alternative causes [91, 93]. Timely recognition and withdrawal of tolvaptan is important when managing DILI. We have summarised a potential approach in Fig. 3.

Investigations for other causes, whilst tolvaptan is withdrawn, may include [94, 95]:

- Viral hepatitis: Hepatitis A virus antibody (IgM), Hepatitis B virus surface antigen (Anti-HBs), Hepatitis C virus antibody (with polymerase chain (PCR) if positive), Hepatitis E virus antibody (IgM), Cytomegalovirus (CMV) antibody (IgM), Epstein-Barr virus (EBV) antibody (IgM)
- Autoimmune conditions: Antinuclear antibody (ANA), Antimitochondrial antibody (AMA), Smooth muscle antibody (SMA)
- Iron studies: Ferritin, Transferrin saturation (TSAT)
- Immunoglobulins: IgA, IgG, IgM
- Imaging: Ultrasound

Elevated liver enzymes typically return to normal after stopping tolvaptan and severe DILI is rare [20]. Observable changes in liver enzymes may be delayed as the half-life for ALT is around 2 days and bilirubin is 17–21 days [96]. If clinically warranted, the frequency of blood tests should be adjusted based on healthcare professional’s judgement. In people with ADPKD who develop DILI upon resuming tolvaptan, it should be stopped and re-exposure should not be attempted [31].

## Electrolyte abnormalities

Higher serum potassium levels can occur with tolvaptan due to an acute reduction in extracellular fluid volume [55, 60]. Higher serum sodium levels were observed in the TEMPO 3:4 trial with a mean increase of less than 2.5 mmol/L at the end of the dose-escalation period [20]. In studies reporting real-world clinical experience [97–99] and in the post-hoc analysis of the TEMPO 3:4 trial [100], a reduction in body weight was observed alongside mild elevations in sodium levels, with dehydration suggested as the underlying mechanism for hypernatremia. In such cases, healthcare professionals are advised to assess the individual’s fluid status if hypernatremia is identified during monitoring and to encourage an increase in fluid intake. The committee is not aware of any reports of rapid overcorrection of hyponatraemia. This is unlikely since there is a warning against using tolvaptan in people with serum sodium abnormalities and it is recommended that this is corrected before tolvaptan initiation [54, 59].

## Urinary retention

Increased fluid intake necessitated by tolvaptan therapy may result in acute urinary retention in the presence of urinary outflow obstruction e.g. prostatic hypertrophy [101]. A clinical history suggestive of retention should be investigated by conducting a post-void bladder scan and referral to local urology services prior to starting tolvaptan. In these scenarios, a risk-benefit assessment should be undertaken as part of the shared decision-making process.

## Other side effects

Urate levels can rise following the initiation of tolvaptan [73]. However, this is rarely significant in clinical practice. In cases of recurrent gout, we would recommend introducing dietary changes and starting allopurinol. If recurrent gout persists, it may be appropriate to consider stopping Tolvaptan.

In the TEMPO 3:4 trial, a higher number of people with ADPKD who received tolvaptan reported skin cancer compared to placebo, predominantly basal cell carcinoma (0.8% versus 0.2%) [20]. However, a causal relationship has not been established and the incidence was less frequent in the TEMPO 4:4 trial [102]. At present there is insufficient evidence to recommend routine monitoring or screening for people taking tolvaptan in the absence of clinical signs or symptoms of skin cancer.

In the TEMPO 3:4 trial, there was an unexpected signal in the adverse events for glaucoma amongst people who received tolvaptan compared to placebo (0.7% versus 0.4%) [20]. Following the trial, Otsuka engaged an independent expert in ophthalmology who did not identify a clear or consistent pattern that would attribute these events to tolvaptan [102]. For this reason, we do not recommend any changes to the NHS recommendation that the general population have their eyes checked every 2 years by an optician. This frequency may be adjusted based on the detection of abnormalities or as advised by their optician or ophthalmologist [103].

## Considerations from a patient perspective

*Recommendation 27*: *Based on patients’ perspectives of tolvaptan we suggest that the following factors should be taken into account when Tolvaptan is being considered – impact on lifestyle*,* occupation*,* family planning*,* healthcare appointments.*

*Recommendation 28*: *When discussing Tolvaptan with patients*,* it is recommended to provide a balanced overview of the positives and negatives.*

Best practice involves making the decision to initiate tolvaptan through a shared decision-making process. This may include the perspectives of the individual with ADPKD, family members, carers, and healthcare professionals from different clinical backgrounds. When discussing potential side effects, it is important to try and present a balanced overview as well as discuss ways to help manage them. Some patients report that it is helpful to have supporting documents from their medical team to help them advocate for themselves, especially early in their journey on Tolvaptan. Effective communication between the medical team and people with ADPKD about potential side effects is crucial. A balanced presentation of tolvaptan’s benefits and side effects is essential, as both people with ADPKD and healthcare professionals can be influenced by framing effects related to timing, lifestyle, employment and healthcare adjustments [104–106].

Positives that might be discussed with people with ADPKD will likely include the slowing of kidney function decline in those with rapid progression [20, 24], delaying the onset of kidney replacement therapy [107], and reducing kidney pain and infections [31]. Negatives discussed with people with ADPKD will likely include an increased thirst and polyuria [20, 24], the risk of liver injury [23], and the need for more monitoring appointments and blood tests.

The following advice has been provided in conjunction with expert patient opinions and those from the Tolvaptan community support group.

### Starting the journey with tolvaptan

When people start taking tolvaptan therapy they often experience almost immediate changes in urinary frequency and thirst. Starting tolvaptan on a day when not at work and travel is not requires can ease the adjustment period [31]. Many people report that, over time – typically days to months – they can return to a near-normal lifestyle as they adapt to the aquaretic side effects. However, some may find that the time is not right to start or continue taking tolvaptan due to a limited ability to manage the side effects or attend the regular appointments e.g. temporary mobility issues or carer responsibilities. Additionally, those of childbearing age may opt to delay treatment until they have completed their family. People with ADPKD should be given the opportunity to restart tolvaptan in the future if they remain eligible when reassessed.

### Tips for dosing and staying hydrated

To maximise comfort, people should take the morning dose of tolvaptan as early as possible, followed by the second dose 6–8 h later, ideally before 5pm. A pragmatic approach to the Otsuka timing 30 min before the morning meal should be taken. Provided that the meal is not a high-fat meal, such as a full English breakfast, this dose may be taken with or without food. This is important given the already significant impact on sleep patterns. Fluid intake should primarily consist of water to avoid excessive calories from sugary or fatty drinks. Suggestions for increasing hydration include using cold infused teabags or sparkling water to combat thirst especially during hot weather where water loss can be higher. When drinking alcohol, it is important that people taking tolvaptan drink more water than usual as alcoholic beverages may reduce thirst sensation. People taking tolvaptan should aim to match their fluid intake with their urine output, which averages around 6–8 L per day, and spread their fluid intake throughout the day and night. People taking tolvaptan should be informed of the indicators of dehydration (e.g. dizziness, light-headedness and pain) and be aware this means they need to drink more water.

### Missing or skipping doses

Discussing “drug holidays” with patients can provide some flexibility during periods of limited restroom access e.g. long journeys or holidays. However, frequent or prolonged “drug holidays” should be discouraged to ensure optimal treatment efficacy.

### Eating well to manage side effects

Dietary adjustment might help with side effects. People taking tolvaptan should be advised to minimise processed food and takeaways, cooking from scratch where possible to reduce salt intake. Some people taking tolvaptan report that high salt intake, especially later in the day, can impact their thirst and aquaretic side effects. Eating a larger meal at lunchtime and lighter meals in the evening has been reported by some people taking tolvaptan to help reduce water consumption overnight. Additionally, some people report that reducing red meat intake and having a lighter meal in the evening can lessen water consumption overnight.

### Getting a good night’s sleep

To improve sleep quality, people taking tolvaptan should keep ample water accessible at their bedside (around 2 L). Earlier bedtimes should be encouraged given the impact of nocturia. Good practices when attending the bathroom overnight to make it easier to return to sleep could include avoiding turning on lights and looking at clocks, watches or mobile phones.

### Navigating daily life

Carrying a letter from their nephrologist, the most senior possible, explaining the need to carry at least two litres of water and access to bathrooms can be helpful, particularly in the workplace or at concerts/theatres/sporting events. The UKKA has a copy of such a letter on the online version of this UKKA commentary. For long-distance travel, it is suggested that people taking tolvaptan can miss tolvaptan doses on the day of the trip as it can be difficult to manage hydration and bathroom breaks. When planning long journeys, people taking tolvaptan should schedule regular stops and consider wearing short, skirts or dresses while driving as this can facilitate easier bathroom access during traffic jams. Keeping wide-opening containers, like old sports water bottles, in the car can help manage emergencies discreetly with other items such as paper towels, a bucket or blankets for privacy. People taking tolvaptan are encouraged to purchase a “Radar key” for access to disabled toilets and obtain a “Just can’t wait” card from the Bladder and Bowel community for quick access to bathroom facilities when needed.

### Being aware of drug interactions and implications for medical care

People taking tolvaptan should have a plan of who they should contact outside of clinic times should they develop a new symptom or they are unsure about a potential drug interaction. Given the specialist nature of tolvaptan, it is unlikely that their community healthcare team will be able to manage this alone. People taking tolvaptan should maintain an updated list of contraindicated medications and food. If temporary medications that interact with tolvaptan are required, such as antibiotics, it may be appropriate to withhold tolvaptan during their use. Prior to taking any herbal remedies or supplements, people taking tolvaptan should consult their medical team. When undergoing surgery that needs general anaesthesia, tolvaptan should be stopped for 3–4 beforehand to minimise the risk during fasting. While the side effects described by manufacturers originate from clinical trials, people taking tolvaptan should be aware that if they develop new symptoms, they should contact their medical team. People taking tolvaptan should attend regular dentist appointments to monitor their gum health as tolvaptan can lead to severe dry mouth. A summary of key information should be given to people taking tolvaptan in the form of a local patient information leaflet or manufacturer leaflet should this not exist. Where possible appointments should be supported through drop-in or drive-through phlebotomy services and remote appointments in virtual or telephone clinics.

### Balancing work and health

People taking tolvaptan need to attend regular appointments, which can affect their employment, finances and overall quality of life. They should be aware of local and national support e.g. social services, Kidney Care UK grants. Despite protections under the Equality Act 2010 [108], people with ADPKD may face discrimination in the workplace. These issues stem from the often invisible nature of ADPKD and unawareness of the effects of tolvaptan [17]. While some adjustments like access to water and bathroom facilities, are relatively straightforward, others such as nocturia affecting sleep quality may be less obvious and particularly challenging for jobs requiring focus, like driving or operating heavy machinery. Patients may benefit from a letter addressed to their employer from their nephrologist, the most senior possible, which explains the need for regular water access and toilet facilities.

Reasonable workplace adjustments could include: (i) Ensuring easy access to refreshment facilities such as water in the workplace, (ii) Ensuring bathroom facilities are easily accessible and there is no restriction on “bathroom breaks”, (iii) Allowing employees to have flexible working hours if they experience nocturia that affects the quality and duration of their sleep, (iv) Doing things another way such as allowing someone on Tolvaptan to have their desk nearer to the bathroom facilities instead of hot-desking.

## Recommendations for kidney units managing ADPKD and initiating tolvaptan

*Recommendation 29*: *Based on the perspectives of the members of the committee working group we suggest the following*:

- *Initial assessment and follow-up in a dedicated genetic/ADPKD/tolvaptan clinic where possible*
- *Multidisciplinary team input*
- *An established pathway for patients with ADPKD on tolvaptan to interact with their kidney unit outside of clinic times*
- *A three-dimensional scan should be performed as part of the initial assessment of a person with ADPKD*
- *All people with ADPKD should be offered genetic testing where available and appropriate as this could inform patient eligibility for tolvaptan*
- *We encourage the registration and monitoring of people with ADPKD through the UKKA RaDaR registry*

In a 2018 survey on tolvaptan prescribing practices in the UK, 93% (41 of 44 centres) of the kidney centres surveyed used tolvaptan in the management of people with ADPKD. Tolvaptan was delivered by a mixture of multi-disciplinary teams, a single responsible clinician and multiple independent clinician models. Assessment methods for tolvaptan eligibility in the responding kidney centres included eGFR slope (100%), mean US kidney length (82%), MRI TKV (53%) and genotype (24%). It is important to note that this survey did not include responses from all kidney units in the UK [109]. Since this survey, the landscape of ADPKD management has changed through the more widespread availability of genetic testing in the NHS and the increasing availability of automated methods for measuring TKV [110].

In this section of the commentary, we describe the best practices in the provision of tolvaptan to people with ADPKD in the UK.

## Healthcare setting

As mentioned in the aforementioned survey, there exists a variety of models used in the delivery of tolvaptan as part of ADPKD care [109]. We recommend that best practice is likely to involve an initial assessment and follow-up undertaken in a kidney unit located at a secondary or tertiary centre [13]. Ideally, this should be under the supervision of nephrologists with a special interest in ADPKD. We recommend that care, where possible, should be multi-disciplinary in nature.

## Imaging

The same survey demonstrated that not all kidney units had access to MRI assessment [109]. We recommend that where possible, an initial three-dimensional MRI or CT scan should be performed as part of the initial assessment of a person with ADPKD in order to have a reliable baseline radiological evaluation of the kidneys and to obtain a total kidney volume (TKV). Considering the small changes in kidney volume between follow-up appointments, volumetric evaluation should not be performed more frequently than every 12 months unless clinically indicated. The increasing availability of automated methods for measuring TKV should make this easier to obtain in the future [110].

## Genetic screening

In the aforementioned survey, it was indicated that a limited number of kidney units had access to genetic testing [109]. However, this is likely to have changed given the widespread availability of genetic testing now. All people with ADPKD should be offered genetic testing where appropriate. Genetic testing has the potential for an earlier diagnosis to inform lifestyle changes, initiation of tolvaptan, family planning and living donation information. However, there are potential important legal, insurance and psychosocial issues that can arise from a genetic diagnosis that should be discussed with people suspected of having ADPKD and those close to them prior to testing [17].

## RaDaR

The National Registry of Rare Kidney Diseases (RaDaR) was developed to collate information from people with certain rare kidney diseases. There are potential benefits for the individual with ADPKD as well as the ADPKD community as a whole for patients to be registered with RaDaR. These include access to relevant information, the potential to contribute to new knowledge, become involved in research studies and attending information events. We recommend that people with ADPKD who are interested should be directed to the RaDaR website.

## Conclusion

This commentary offers comprehensive and practical guidance on optimising tolvaptan use in ADPKD care. By integrating up-to-date evidence, real-world insights and patient perspectives, we present actionable recommendations that emphasise patient-centred approaches to optimise outcomes. Through close attention to eligibility criteria, side effect management and patient education, a holistic framework emerges that facilitates adherence and enhances quality of life. Balancing medical guidance with the lived experiences of patients leads to adaptable, practical strategies for initiating tolvaptan, ultimately driving better care for people with ADPKD.

## Data Availability

No datasets were generated or analysed during the current study.

## Abbreviations

ACEi: Angiotensin Converting Enzyme inhibitor
ADPKD: Autosomal Dominant Polycystic Kidney Disease
ANA: Antinuclear Antibody
AMA: Antimitochondrial Antibody
ARB: Angiotensin Receptor Blocker
BNF: British National Formulary
CAMP: Cyclic adenosine monophosphate
CRISP: Consortium for Radiologic Imaging Study of PKD
CT: Computed Tomography
DILI: Drug Induced Liver Injury
eGFR: Estimated Glomerular Filtration Rate
MRI: Magnetic Resonance Imaging
NHS: National Health Service
NICE: National Institute for Health and Care Excellence
PCR: Polymerase Chain Reaction
PROPKD: Predicting Renal Outcomes in ADPKD
REPRISE: Replicating Evidence of Preserved Renal Function: an Investigation of Tolvaptan Safety and Efficacy in ADPKD
SGLT2i: Sodium-Glucose Transport Protein 2 inhibitors
SMA: Smooth Muscle Antibody
SMC: Scottish Medicines Compendium
SPC: Summary of Product Characteristics
TEMPO: Tolvaptan Efficacy and Safety in Management of Autosomal Dominant Polycystic Kidney Disease and its Outcomes
TKV: Total Kidney Volume
TSAT: Transferrin Saturation
US: Ultrasound
